# Semi-supervised Bayesian deep clustering with uncertainty-guided representation and robustness

**DOI:** 10.3389/frai.2026.1935933

**Published:** 2026-10-05

**Authors:** S. Krishnamoorthy, B. Jaganathan

**Affiliations:** Department of Mathematics, School of Advanced Sciences, Vellore Institute of Technology (VIT), Chennai, India

**Keywords:** Bayesian deep clustering, clinical risk stratification, Gaussian mixture prior, missing data, robust representation learning, semi-supervised learning, Student-t likelihood

## Abstract

**Introduction:**

In real-world data, tackling missing data, noisy observations, and uncertainty at the same time is hard since the number of labelled data is limited. Current deep clustering algorithms have considered the imputation, noise, semi-supervision, and uncertainty problems separately, such as without modelling the uncertainty in the noise, or without modelling the missingness in the missing data. There is still a need to develop an end-to-end deep clustering algorithm that incorporates all these four factors.

**Methods:**

To overcome the limitations, we propose to robust the semi-supervised Bayesian Deep Clustering (RBDC), which involves four parts, namely: The Student-t decoder does not explicitly impute for missing features and yields a robust reconstruction for heavy-tailed noise. Label-guided Gaussian mixture priors provide partial class label guidance in the learning process within the latent space, which is positioned between supervised and unsupervised learning. An uncertainty-weighted Evidence Lower Bound (ELBO) scales the contribution of each sample by the posterior uncertainty, effectively being noisy or contaminated. Sytems with Hybrid EM + SGD optimisation strategy. The mixture is trained using the hybrid EM + SGD optimisation strategies. The latent uncertainty is used in the mixture updates. The framework is tested on University of California, Irvine (UCI) Adult Income and Gym Members Fitness datasets, and the results are compared against strong deep clustering baselines.

**Results:**

Results show that RBDC outperforms baseline methods in calibration, discrimination/predictive ability and clustering quality in the low supervision regime. Ablation studies suggest the benefit of label informed priors and uncertainty weighting in enabling stable and interpretable clustering. Results also highlight the complementary benefits of semi-supervision and modelling of uncertainty.

**Discussion:**

Our results show that RBDC offers a strong and label-efficient calibration-aware framework to clustering of noisy, incomplete and partially labelled tabular data. RBDC attempts to model in a single generative framework the missingness, noise, uncertainty and weak supervision in the data, with a possible benefit to downstream applications in decision support such as risk stratification, anomaly detection and predictive modelling. Hence RBDC provides a conceptually integrated approach to semi-supervised deep clustering for challenging real-world datasets.

**Subject classification codes: 11Y16, 62H20, 62H30, 68T07, 68T09, 90C52**

## 1 Introduction

A fundamental method for discovering hidden structure in complicated, high-dimensional data, clustering has a wide range of uses, including fraud detection, genomics, clinical risk assessment, and industrial process monitoring. Clustering is crucial, but it's still quite difficult in real-world situations because data is rarely clean, comprehensive, or completely unlabeled. The majority of classical and deep clustering techniques perform poorly on practical datasets, which are often contaminated by noise, burdened by missing entries, and characterized by a sparse yet highly informative set of class labels. Classical methods such as k-means and Gaussian mixture models (GMMs) are inherently vulnerable to violations of these requirements, as they produce deterministic, hard-cluster assignments and assume fully observed, noise-free data (Wang and Lin, 2022). By simultaneously learning latent representations and cluster assignments, deep clustering techniques such as Variational Deep Embedding (VaDE) (Jiang et al., 2017) and Deep Embedded Clustering (DEC) (Xie et al., 2016) extend clustering into the deep learning paradigm. Variational techniques using Gaussian mixture priors (GMVAE) (Dilokthanakul et al., 2017) and non-Gaussian priors (Guo et al., 2025) have shown strong performance on benchmark datasets. Three co-occurring issues that arise in high-stakes applications, however, are not fully addressed by even these more potent techniques: (i) systematic robustness to outliers and heavy-tailed noise; (ii) principled propagation of epistemic uncertainty throughout the pipeline; and (iii) the efficient use of partial supervision to anchor latent cluster structure.

To increase cluster coherence, pairwise restrictions (Basu et al., 2004) or partial labels (Hsu and Kira, 2015; Kingma et al., 2014) have been incorporated into semi-supervised clustering techniques. Generative priors can encode label information, as shown by Kingma et al.'s (2014) semi-supervised Variational Auto-Encoder (VAE), while more recent adaptive deep clustering methods (Chang et al., 2017) have improved representations using different supervision signals. Concurrently, MC-dropout (Gal and Ghahramani, 2016), deep ensembles (Lakshminarayanan et al., 2017), and variational formulations (Kendall and Gal, 2017; Nicholls et al., 2024) have advanced uncertainty quantification in Bayesian deep learning. However, current approaches either quantify uncertainty without utilizing it to regulate learning and downstream prediction or use semi-supervision without principled uncertainty propagation. As a result, these lines of inquiry have developed mainly independently. There is a crucial, unexplored gap where these three requirements intersect. None of the aforementioned paradigms by themselves offer a workable solution when data are concurrently noisy, partially observed, and partially labeled, as is common in sensor networks, clinical trial data, and electronic health records. Specifically, existing frameworks do not generate calibrated, uncertainty-adjusted cluster memberships that can serve as reliable features for downstream decision support, nor do they systematically leverage the variational encoder's posterior uncertainty to weight sample contributions during cluster parameter estimation. The lack of a single probabilistic design concept that jointly models noise, missingness, and partial supervision as distinct preprocessing issues rather than as separate modeling concerns is the reason for this gap, which is not just architectural.

Diffusion-based latent models and graph-integrated Bayesian frameworks are two recent developments that have suggested richer inductive biases for representation learning. Although these approaches show promise, they entail high architectural and computational costs that may be inappropriate for tabular clinical data, where label efficiency, interpretability, and calibration are critical. Therefore, the main question driving this work is not whether a more complex model can be built, but rather whether robustness, uncertainty awareness, semi-supervised guidance, and downstream predictive utility can be achieved simultaneously within a tractable, theoretically grounded optimization procedure that leverages a principled, unified probabilistic framework.

Although Student-t likelihoods, mixture VAEs, semi-supervised VAEs, missing-data VAEs, and uncertainty-aware clustering have been explored individually, it is the synchronized, synergistic integration within a single training objective that constitutes the Robust Bayesian Deep Clustering (RBDC's) uniqueness. Label-guided priors inform the mixture topology, which is subsequently down-weighted for each sample through the uncertainty-weighted ELBO. The Student-t likelihood for robustness and EM safeguard against posterior covariance collapse are both informed by the same shared posterior covariance estimates. No previously published semi-supervised or robust VAE-clustering method has integrated uncertainty propagation, label anchoring, and heavy-tailed robustness simultaneously using a single, shared posterior covariance term—it is this integration, not the disparate component models that RBDC exploits, which we assert is novel.

Accordingly, we propose the Robust Bayesian Deep Clustering (RBDC) framework, a semi-supervised, uncertainty-aware Bayesian deep clustering model that addresses these challenges through four key contributions:

**(i) Label-Informed Gaussian Mixture Priors:** RBDC incorporates limited class labels directly into the Gaussian mixture prior in latent space, enabling even small amounts of supervision (e.g., 1% labeled data) to guide cluster formation in a principled probabilistic manner.

**(ii) Uncertainty-Weighted ELBO:** We introduce an uncertainty-weighted ELBO where each sample's contribution is scaled inversely to the encoder posterior covariance, reducing the impact of uncertain or corrupted samples during training.

**(iii) Hybrid EM** **+** **SGD Optimization:** RBDC combines end-to-end gradient training with uncertainty-aware EM-style updates for Gaussian mixture parameters, enabling stable and interpretable clustering.

**(iv) Uncertainty-Calibrated Prediction:** In conjunction with latent embeddings, the framework produces uncertainty-aware soft cluster memberships that enhance calibration and dependability in subsequent tasks, including risk stratification, diagnosis, and anomaly detection.

The remainder of this paper is organized as follows: Section 2 reviews related work across five interlocking bodies of literature. Section 3 presents the full RBDC methodology with step-by-step derivations. Section 4 reports experimental results on both datasets, including ablation studies and significance tests. Section 5 concludes with a discussion of limitations and future directions, including planned extensions to graph-structured and high-dimensional clinical benchmarks.

## 2 Literature review

The development of the RBDC framework draws on five interrelated bodies of literature—classical and robust mixture models, deep clustering and variational autoencoders, techniques for noisy and incomplete data, semi-supervised clustering, and Bayesian uncertainty quantification. We examine each strand, pinpoint its shortcomings in the joint regime of noise, missingness, and partial supervision, and compare RBDC to the most cutting-edge techniques.

Due to their analytical tractability and interpretability, K-means and Gaussian mixture models (GMMs) remain the most popular baselines for clustering (Wang and Lin, 2022). The Expectation-Maximization (EM) algorithm, which alternates between closed-form parameter updates and soft cluster assignment, is used to optimize both approaches. Both, however, are vulnerable to non-Gaussian cluster geometries, initialization sensitivity, and the existence of outliers or heavy-tailed noise—limitations that are extensively described in the statistical literature (Wang and Lin, 2022). Strong mixture models, especially those based on the Student-t distribution (Peel and McLachlan, 2000), were developed expressly to reduce the impact of extreme observations by assigning heavy-tailed probability mass to distant data points. The degrees-of-freedom parameter v. The Student-t determines the tail weight converges to a Gaussian as ω → ∞, whereas modest σ (e.g., σ = 3–5) offers strong outlier suppression. By combining robust likelihoods with low-rank covariance structures, t-factor analysers (Wang and Lin, 2022) fill in the gaps in the mixture modeling paradigm, while extensions to skew-t mixtures (Wei et al., 2019) further accommodate asymmetric cluster configurations. These techniques serve as the statistical basis for the Student-t decoder design used by RBDC. The nonlinear, high-dimensional representations that characterize contemporary clinical or sensor data cannot be learned by standard mixture algorithms, despite their robustness. Instead, they operate on predefined, manually constructed feature spaces. This encourages their integration with deep representation learning.

The combination of autoencoders and clustering was first demonstrated by Deep Embedded Clustering (DEC) (Xie et al., 2016), which used self-training with Student-t soft assignments to optimize a reconstruction objective and a soft cluster-assignment target concurrently. Although powerful, DEC assumes clean, fully observed data and executes deterministic assignment without quantifying uncertainty. A Gaussian mixture prior was then added to the VAE latent space using Variational Deep Embedding (VaDE) (Jiang et al., 2017), thereby enabling simultaneous generative modeling and soft cluster-structure learning. The strongest standard by which RBDC is measured is VaDE. Gamma mixture VAEs (Guo et al., 2025) showed more flexibility for non-Gaussian cluster forms on both image and tabular benchmarks, while Gaussian Mixture VAEs (GMVAE) (Dilokthanakul et al., 2017) expanded this method with a principled variational formulation. Graph-integrated techniques have become a potent complement to VAE-based deep clustering in recent years. The relational structure among data points significantly enhances cluster coherence in heterogeneous data, as shown by multi-view graph clustering with graph-regularized matrix factorization (Li and Li, 2026). Wu et al. (2020) describe the general field of graph neural networks, of which jointly learning the clustering assignments and the graph topology is an exciting area. Parallel work in tensor-constrained Multiview subspace clustering (Zhang et al., 2015) demonstrates that incorporating relational structure between views increases cluster consistency in heterogeneous multi-source data, suggesting a similar avenue for applying the graph structure in conjunction with partial labels. These graph-based techniques constitute a significant and rapidly expanding field that has yet to be explored by purely latent-variable models, such as RBDC. This acknowledged restriction is revisited in Section 5. The assumption of clean, fully observed data during training is a crucial flaw shared by DEC, VaDE, and GMVAE. The primary gap that RBDC addresses is the lack of a defined method for propagating uncertainty from noisy or missing observations through the clustering pipeline.

In deep generative models, handling missing data remains a significant challenge. Imputed values are treated as observed data by common preprocessing techniques such as mean imputation, MICE, and GAIN (Yoon et al., 2018), thereby ignoring uncertainty and potentially introducing bias into downstream grouping and prediction. According to Nazabal et al. (2020), latent representations are improved, and uncertainty is preserved when missingness is explicitly incorporated into the VAE likelihood. By evaluating the Student-t reconstruction likelihood solely on observable dimensions and eliminating the imputation phase, RBDC natively handles missing data in accordance with this approach. Compared with Gaussian decoders, scale-mixture formulations and Student-t likelihoods in deep latent variable models (Zhao et al., 2017) have shown greater robustness to outliers and corrupted observations. Nevertheless, resilient decoders of this type have rarely been integrated with principled uncertainty propagation and semi-supervised guidance within a unified clustering framework; RBDC directly closes this gap.

Constraint-based techniques that employ must-link and cannot-link information to direct clustering are part of semi-supervised clustering. Such limitations were incorporated into clustering targets in early probabilistic (Basu et al., 2004), kernel-based (Kulis et al., 2005), and deep learning (Hsu and Kira, 2015) techniques. However, these approaches are limited in their ability to directly modify the latent-space structure, as they affect cluster assignments rather than the underlying generative latent distribution. Labels are directly incorporated into the probabilistic model in generative semi-supervised approaches. Latent class variables that use both labeled and unlabeled data were first proposed by Kingma et al. in their semi-supervised VAE (Kingma et al., 2014). More recent methods (Arazo et al., 2020; Becker and Kohavi, 1996) expand on this concept by using teacher-student frameworks and pseudo-labeling to improve latent representations gradually. RBDC expands the generative label-incorporation paradigm in two significant ways. Instead of introducing a distinct classification head, labels are first added at the level of the Gaussian mixture prior, fixing the prior component for labeled points. Second, to prevent low-quality or corrupted labeled instances from dominating the ELBO objective, the encoder posterior's uncertainty is explicitly used to adjust the influence of each labeled and unlabeled sample during training.

In Bayesian deep learning, uncertainty quantification has become increasingly important, especially for safety-critical applications in industrial monitoring and healthcare. Aleatoric uncertainty, or irreducible noise present in the data, is distinguished from epistemic uncertainty, or model uncertainty reducible with more data, according to a fundamental distinction made by Kendall and Gal (2017). For dependable downstream decision support, both types of uncertainty need to be modeled: aleatoric uncertainty reflects the intrinsic noisiness of predictions. At the same time, epistemic uncertainty provides information when the model lacks sufficient training data. Common techniques for estimating uncertainty include variational Bayesian approaches (Kendall and Gal, 2017; Nicholls et al., 2024), which offer principled posterior inference at a higher computational cost; deep ensembles (Lakshminarayanan et al., 2017), which estimate uncertainty via prediction disagreement across multiple models; and MC-dropout (Gal and Ghahramani, 2016), which approximates Bayesian inference via stochastic forward passes. Nicholls et al. (2024) demonstrated that explicitly modeling assignment uncertainty in clustering, rather than using hard or soft assignments, enhances cluster stability and interpretability under incomplete data. In a similar vein, Nazabal et al. (2020) showed that propagating uncertainty through the encoder enhances representation and imputation quality in heterogeneous-data VAEs. Despite recent advancements, clustering techniques still lack a systematic way to generate uncertainty-calibrated cluster memberships for subsequent tasks and to use encoder posterior covariance to weight sample contributions during parameter estimation. To overcome this, RBDC leverages encoder uncertainty across all aspects of the framework, including downstream feature generation, the training target, and the EM M-step.

Besides the graph-integrated and generative models discussed so far, a recent wave of architectures since 2024 has further diversified the space of deep clustering. Attention-based and transformer components have been incorporated more deeply into clustering objectives; Ren et al. (2025) provide an extensive survey of this rapidly growing area of self-supervised, generative, and attention-driven clustering, placing these methods in a unified taxonomy that covers both image and tabular data. In the graph learning domain, modularity-maximizing neural methods such as DGCluster (Bhowmick et al., 2024) employ graph neural networks to optimize node embeddings and a differentiable community-structure objective simultaneously, thereby extending previous graph-regularized techniques to enable entirely end-to-end trainable clustering. Recently, diffusion-based generative models have been explored for clustering: score-based and denoising-diffusion methods, such as Clustering via Diffusion (CLUDI) (Uziel et al., 2025), frame cluster assignment as an amortized, self-supervised inference task over already-learned representations, thereby outperforming prior work without requiring the number of clusters to be fixed beforehand. While these transformer-, graph-, and diffusion-based models enhance representation variety and provide an alternate, non-parametric path to cluster finding in the case of diffusion, they often lack emphasis on calibrated uncertainty propagation, semi-supervised label anchoring, and performance on heavily-tailed noise in low-dimensional tabular data—the three features on which RBDC is designed to achieve joint high performance. Rather than competing on representational flexibility, it is most useful to view RBDC as an accompanying, interpretable Bayesian method tailored to this noisy, partially labeled tabular data regime on which these more complex architectures seldom succeed.

Some recent works focus on subsets of the RBDC properties—semi-supervision, robustness, and uncertainty—and some of these are quite close to the specific combined methodology of RBDC—for instance, Qin et al. (2019). Present a semi-supervised deep clustering approach utilizing pairwise constraints and sample similarity to steer representation learning, but do not provide a probabilistic framework for uncertainty or resilience against heavy-tailed noise; Eduardo et al. (2020). Develop a robust VAE that handles outlier detection and imputation of mixed-type data by jointly modeling robustness and missingness. Still, it doesn't anchor the uncertainty with semi-supervision or apply an uncertainty-weighted loss function to the objective. Kim et al.'s (2024). t-VAE extends the VAE to use t-distributions throughout the network for robustness to heavy tails, sharing the idea of Student's t-distribution in the decoder as with RBDC, but they do not exploit semi-supervision, and their objective is not for clustering; Uncertainty-Based Learning for Deep Clustering (ULDC) is a deep cluster that explicitly models prediction uncertainty to guide the clustering objective, but not by anchoring uncertainty to partial label information or by propagating uncertainty through a Bayesian mixture before its use as a learning objective. By optimizing a unique posterior covariance for all three semi-supervised label information, robust Student's t sampling, and uncertainty-weighted optimization, RBDC directly integrates these into a joint objective, unlike other methods that address each component in isolation.

While mixed models rely on EM with closed-form updates, deep clustering techniques typically use Stochastic Gradient Descent (SGD) with backpropagation. Hybrid EM–SGD techniques, such as VaDE (Jiang et al., 2017), combine closed-form mixture updates with neural network training to increase stability. By adding encoder posterior covariance to the goal function and to the M-step updates, RBDC extends this approach. RBDC reduces covariance collapse and produces cluster parameters that more accurately reflect the data distribution by accounting for both latent dispersion and uncertainty, unlike conventional approaches that treat latent means as point estimates. While some of the issues discussed here are addressed by techniques such as GMVAE (Dilokthanakul et al., 2017) and the semi-supervised VAE (Kingma et al., 2014), no method currently in use simultaneously achieves all five features. Emerging diffusion-based and graph-integrated Bayesian frameworks provide richer inductive biases for structured and relational data. Still, they entail significantly greater architectural complexity and lack the interpretable semi-supervised calibration qualities that RBDC seeks. As a tractable, theoretically sound, and label-efficient Bayesian clustering framework optimized for the high-noise, high-missingness, low-label regime of tabular clinical and engineering data, RBDC fills a clear and distinct niche.

## 3 Methodology

The proposed Robust Bayesian Deep Clustering (RBDC) framework is described in detail in this section. To represent the entire modeling process, the presentation is arranged in sequence. The problem formulation and data structure are presented first, followed by the specification of the generative model and the derivation of the variational inference objective. The uncertainty-weighting process and the addition of semi-supervised data are then explained. This part explains the construction of the hybrid optimization technique, the extraction of latent representations for downstream prediction tasks, and the final training process. This structure ensures a logical flow of concepts and makes it easier to understand the mathematical underpinnings of the proposed framework.

### 3.1 Generative model

Consider a dataset consisting of N observations, where each data point x_*i*_ ε R^*D*^ may be only partially observed. Let O_*i*_ ⊆ {1, 2, …, D} denote the set of observed feature indices for sample *i*. Any feature dimension d ε O_*i*_ is regarded as missing and is excluded from likelihood evaluation; no missing-value imputation is performed. The dataset is partitioned into a labeled subset L = {*i*: y_*i*_ is known} and an unlabeled subset U = {*i*: y_*i*_ is unknown}, where y_*i*_ ε {1, 2, …, C} denotes the class label.

To represent the latent structure of the data, each observation is associated with a continuous latent variable z_*i*_ ε R^d^ and a discrete cluster assignment c_*i*_ ε {1, 2, …, *K*}.

#### 3.1.1 Cluster assignment prior

The cluster assignment variable follows a categorical distribution parameterized by mixture weights π = (π1, π_2_, …, π_K_), satisfying Σ_*k*_π_*k*_ = 1:


$$\begin{array}{l} \text{p}\left({\text{c}}_{\text{i}}=k\right)=\pi_{\text{k}}\ldots \end{array}$$ (1)

#### 3.1.2 Cluster-conditional latent prior

Given cluster assignment *c*_*i*_ = *k* in Equation 1, the latent representation is drawn from a Gaussian distribution with cluster-specific mean μ_*k*_ and covariance Σ_*k*_ as specified in the Equation 2:


$$\begin{array}{l} \text{p}\left({\text{z}}_{\text{i}}|c_{i}=k\right)=\text{N}\left({\text{z}}_{\text{i}};\mu_{k},\Sigma_{k}\right) \end{array}$$ (2)

The parameters {π_*k*_, μ_*k*_, Σ_*k*_}; *k*=*1,2,…, K* are learned during training using the uncertainty-aware EM update procedure.

Marginalizing over all possible cluster assignments yields the latent prior


$$\begin{array}{l} \text{p}\left({\text{z}}_{\text{i}}\right)={\text{Σ}}_{k=1}^{K}\pi_{k}N\left(z_{i};\mu_{k},\Sigma_{k}\right) \end{array}$$ (3)

which corresponds to a Gaussian Mixture Model (GMM) in latent space.

#### 3.1.3 Student-t observation likelihood

Given the latent representation *z*_*i*_, the observed feature indices $O_{i}$ into a continuous subset $O_{i}^{count}$ are partitioned and a categorical subset, grouped by the original categorical variable *j*∈{1, …, *J*} with *L*_*j*_ levels each rather than treating each one-hot column independently. The likelihood of the continuous dimensions is


$${\text{p}}_{\theta }({\text{x}}_{\text{i},O_{\text{i}}}|{\text{z}}_{\text{i}})=\prod_{\text{d}\in O_{\text{i}}}{\text{Student-t(x}}_{\text{i},\text{d}};\mu_{\text{d}}({\text{z}}_{\text{i}}),\sigma_{\text{d}}^{\text{2}}({\text{z}}_{\text{i}}),\nu )$$ (4)

where μ_*d*_(*z*_*i*_) and $\sigma_{d}^{2}\left(z_{i}\right)$ are the decoder-predicted mean and variance, respectively, and ν is the degrees-of-freedom parameter.

The corresponding log-likelihood for a single observed feature is


$$\log{\text{p}}_{\theta }({\text{x}}_{\text{i},\text{d}}|{\text{z}}_{\text{i}})\;=\log\Gamma \left(\frac{\nu \text{+1}}{\text{2}}\right)-\;\log\Gamma \left(\frac{\nu }{\text{2}}\right)-\frac{\text{1}}{\text{2}}\log(\pi \nu \sigma_{\text{d}}^{\text{2}}({\text{z}}_{\text{i}}))-\frac{\nu \text{+1}}{\text{2}}\log\left(\text{1+}\frac{{\left({\text{x}}_{\text{i},\text{d}}\text{-}\mu_{\text{d}}({\text{z}}_{\text{i}})\right)}^{\text{2}}}{\nu \sigma_{\text{d}}^{\text{2}}({\text{z}}_{\text{i}})}\right)$$ (5a)

The likelihood is evaluated only over observed dimensions $\text{d}\in O_{\text{i}}$, naturally handling missing features without imputation.

Each categorical variable *j* is modeled jointly across its *L*_*j*_ levels—not as independent Bernoulli draws per one-hot column, since one-hot levels are mutually exclusive rather than independent. The decoder outputs a categorical distribution via a softmax head:


$$p_{\theta }(x_{i,j}|z_{i})=\text{Categorical}\;(x_{i,j};softmax(g_{j}(z_{i})))$$ (5b)

with log-likelihood


$$\log p_{\theta }(x_{i,j}|z_{i})=\sum_{l=1}^{L_{j}}x_{i,j,l}\log{\left[\text{softmax}(g_{j}(z_{i}))\right]}_{l}$$ (5c)

where *x*_*i, j, l*_ is the one-hot indicator of the variable *j* takes level *l* for sample *i*, and *g*_*j*_(*z*_*i*_) is a small decoder head producing *L*_*j*_ logits. As with the continuous case, categorical variables are not observed for the sample. *i* are excluded from the likelihood without imputation.

#### 3.1.4 Complete joint distribution

Combining the observation model, latent prior, and cluster prior yields the complete-data generative model in Equation 6:


$$\begin{array}{l} {\text{p}}_{\text{θ}}\left(x_{i},z_{i},c_{i}\right)={\text{p}}_{\text{θ}}\left(x_{i,O_{i}}|z_{i}\right)p\left(z_{i}|c_{i}\right)p\left(c_{i}\right) \end{array}$$ (6)

where *p*(*z*_*i*_∣*c*_*i*_) is the cluster-specific Gaussian prior and *p*(*c*_*i*_) is the categorical cluster assignment prior. The decoder outputs μ_*d*_(*z*_*i*_) and $\sigma_{d}^{2}\left(z_{i}\right)$ contribute to the ELBO reconstruction term, while the Gaussian mixture prior contributes to the KL-divergence terms.

### 3.2 Variational inference and evidence lower bound

The posterior *p*(*z*_*i*_, *c*_*i*_∣*x*_*i*_) is intractable because the latent prior is a Gaussian mixture. From Equation 6 we therefore introduce a variational approximation and optimize an Evidence Lower Bound (ELBO).

#### 3.2.1 Variational posterior

An Multi-Layer Perceptron (MLP) encoder [*D* → 128 → 64 → 32 → *d*] with ReLU activations, it maps the partially observed input $x_{i,O_{i}}$, augmented with a binary missingness mask, to the parameters of a diagonal Gaussian posterior:


$$\begin{array}{l} {\text{q}}_{\text{ϕ}}\left(z_{i}|x_{i},O_{i}\right)=N\left(z_{i};{\hat{\mu }}_{\text{i}},{\hat{\Sigma }}_{\text{i}}\right) \end{array}$$ (7)

where ${\hat{\Sigma }}_{i}=\text{diag}\left({\hat{\sigma }}_{i,1}^{2},\ldots ,{\hat{\sigma }}_{i,1}^{d}\right)$. The posterior covariance ${\hat{\Sigma }}_{i}$ provides the latent uncertainty estimates used throughout the RBDC framework.

Latent samples are obtained using the reparameterization trick:


$$\begin{array}{l} {\text{z}}_{\text{i}}={\hat{\mu }}_{\text{i}}+{\hat{\Sigma }}_{\text{i}}^{1/2}⊙\text{ε},\text{ε}\sim N\left(0,I\right) \end{array}$$ (8)

#### 3.2.2 General ELBO

Applying Jensen's inequality yields


$$\begin{array}{l} \text{log}p_{\theta }\left({\text{x}}_{i}\right)\;\geq E_{q_{\phi }}\left[\text{log}p_{\theta }\left(|{\text{x}}_{i,O_{i}}|{\text{z}}_{i}\right)\;\right]-\text{KL }\left[q_{\phi }\left(|{\text{z}}_{i}|{\text{x}}_{i}\right)||p\left({\text{z}}_{i}\right)\right] \end{array}$$ (9)

The first term measures reconstruction quality under the Student-*t* decoder, while the KL term regularizes the latent representation toward the prior.

#### 3.2.3 Labeled ELBO

For labeled samples (*i*∈*L*), the latent prior is fixed to the class-specific Gaussian $N\left(\mu_{y_{i}},\Sigma_{y_{i}}\right)$:


$$\begin{array}{lll} {ℒ}_{i}^{lab} & = & E_{q_{\phi }}[\log p_{\theta }(x_{i,O_{i}}|z_{i})] \end{array}\begin{array}{l} -KL\;[q_{\phi }(z_{i}|x_{i})∥N(\mu_{y_{i}},\Sigma_{y_{i}})] \end{array}$$ (10)

Since both distributions are Gaussian, the KL divergence is computed analytically:


$$KL=\frac{1}{2}[\log\frac{|\Sigma_{k}|}{|{\hat{\Sigma }}_{i}|}-d+Tr(\Sigma_{k}^{-1}{\hat{\Sigma }}_{i})+{\left({\hat{\mu }}_{i}-\mu_{k}\right)}^{T}\Sigma_{k}^{-1}({\hat{\mu }}_{i}-\mu_{k})]$$ (11)

#### 3.2.4 Unlabeled ELBO

For unlabeled samples (*i*∈*U*), the prior remains the full Gaussian mixture:


$$\begin{array}{l} p\left(z_{i}\right)=\overset{K}{\underset{k=1}{\sum }}\pi_{k}N\left(z_{i};\mu_{k},\Sigma_{k}\right). \end{array}$$

The corresponding ELBO is


$$\begin{array}{lll} {ℒ}_{i}^{unlab} & = & E_{q_{\phi }}[\log p_{\theta }(x_{i,O_{i}}|z_{i})]+E_{q_{\phi }}[\log\overset{K}{\underset{k=1}{\sum }}\pi_{k}N(z_{i};\mu_{k},\Sigma_{k})] \end{array}\begin{array}{l} +H(q_{\phi }) \end{array}$$ (12)

where the entropy of the diagonal Gaussian posterior is


$$\begin{array}{l} H\left(q_{\phi }\right)=\frac{1}{2}\overset{d}{\underset{j=1}{\sum }}\left[1+\log\left(2\pi {\hat{\sigma }}_{i,j}^{2}\right)\right] \end{array}$$ (13)

The mixture-prior expectation has no closed-form solution and is estimated using *M* = 5 Monte Carlo samples obtained via Equation 8.

#### 3.2.5 Semi-supervised objective

The overall training objective combines labeled and unlabeled contributions:


$$\begin{array}{l} L_{semi}=\underset{i\in L}{\sum }L_{i}^{lab}+\lambda_{u}\underset{i\in U}{\sum }L_{i}^{unlab} \end{array}$$ (14)

where λ_*u*_ controls the influence of unlabeled data. In all experiments, λ_*u*_ = 1.

### 3.3 Semi-supervised responsibilities

Given a latent representation *z*_*i*_, the responsibility of the mixture component *k* is the posterior cluster assignment probability


$$\begin{array}{l} r_{ik}=p\left(c_{i}=k|z_{i}\right)=\frac{\pi_{k}N\left(z_{i};\mu_{k},\Sigma_{k}\right)}{\overset{K}{\underset{j=1}{\sum }}\pi_{j}N\left(z_{i};\mu_{j},\Sigma_{j}\right)} \end{array}$$ (15)

Since z_*i*_ is not directly observed but distributed according to the variational posterior *q*_ϕ_(z_*i*_∣x_*i*_), the exact expected responsibility requires integrating over *q*_ϕ_, which is intractable. We therefore use the approximation


$$\begin{array}{l} r_{ik}\approx \frac{\pi_{k}N\left({\hat{\mu }}_{i};\mu_{k},\Sigma_{k}+{\hat{\Sigma }}_{i}\right)}{\overset{K}{\underset{j=1}{\sum }}\pi_{j}N\left({\hat{\mu }}_{i};\mu_{j},\Sigma_{j}+{\hat{\Sigma }}_{i}\right)} \end{array}$$ (16)

This corresponds to evaluating cluster likelihoods at the encoder mean while accounting for posterior uncertainty through covariance inflation. As ${\hat{\text{Σ}}}_{i}\to 0$, Equation 16 reduces to the standard GMM responsibility in Equation 15. Larger posterior uncertainty yields softer, more diffuse assignments.

For labeled samples (*i*∈*L*), responsibilities are fixed to the known class:


$$\begin{array}{l} r_{i,y_{i}}=1,r_{ik}=0,k\neq y_{i} \end{array}$$ (17)

This hard assignment anchors each mixture component to its corresponding labeled examples and guides the cluster geometry during parameter updates.

Since labeled samples directly anchor the mixture component parameters to the observed values in Equation 17, clustering and predictive metrics calculated on the labeled partition L are prone to being trivially favored by RBDC due to label leakage. L is the labeled subset; we randomly select samples from the training dataset with no explicit class-level separation between known and unknown classes; therefore, all classes could be included in the labeled subset. Ground truth for all test instances is provided and used only during the evaluation phase. The proposed evaluation phase lacks an independent unknown-class test set; the entire test set is utilized only during evaluation, whereas the test data is unseen for anchoring (Equation 17), tuning, pruning, and calibration.

### 3.4 Uncertainty weighting

Samples with high missingness, noise, or ambiguous latent representations typically exhibit larger posterior variance. To reduce their influence during learning, RBDC assigns each sample an uncertainty-dependent weight:


$$\begin{array}{l} w_{i}=\frac{1}{1+\alpha Tr\left({\hat{\Sigma }}_{i}\right)},\alpha >0 \end{array}$$ (18)

where $\text{Tr}\left({\hat{\text{Σ}}}_{i}\right)=\overset{d}{\underset{j=1}{\sum }}{\hat{\sigma }}_{i,j}^{2}$ denotes the total latent posterior variance. Throughout this work, α = 0.5. The weighting function decreases monotonically with uncertainty: *w*_*i*_ → 1 for highly confident samples $\left(\text{Tr}\left({\hat{\text{Σ}}}_{i}\right)\to 0\right)$, and *w*_*i*_ → 0 as uncertainty increases. Since *w*_*i*_ depends directly on the encoder covariance, it remains fully differentiable and can be optimized jointly with the variational parameters.

In this work, we identify three associated yet conceptually distinct definitions of uncertainty: (i) variational posterior variance ${\hat{\Sigma }}_{i}$ (produced by the encoder) which estimates how confident the model is of its own latent representation of sample *i*; (ii) Aleatoric uncertainty due to variations that are inherently random in the features is not explicitly modelled and could be mitigated through Student-t decoder, which has heavy-tails; and (iii) epistemic uncertainty, relating to model uncertainty arising from the scantness of labels. Reducing epistemic uncertainty occurs because many labeled inputs more accurately anchor the mixture components. The mechanism (Equations 18–21) applies weighting of the loss based on measure (i), which represents a tractable proxy for the combined measure of both (ii) and (iii).

The uncertainty-weighted semi-supervised objective is


$$\begin{array}{l} L_{semi}^{w}=\underset{i\in L}{\sum }w_{i}L_{i}^{lab}+\lambda_{u}\underset{i\in U}{\sum }w_{i}L_{i}^{unlab} \end{array}$$ (19)

where λ_*u*_ balances labeled and unlabeled contributions. Equation 19 is the objective optimized during training, while Equation 14 represents its unweighted counterpart.

### 3.5 Downstream predictive integration

After training, the encoder mean ${\hat{\mu }}_{i}$ and estimated cluster parameters are used to construct uncertainty-aware predictive features.

#### 3.5.1 Soft cluster membership

Cluster memberships are computed using a temperature-scaled softmax over Mahalanobis distances:


$$\begin{array}{l} \pi_{ik}=\frac{\exp\left(-d_{M}\left({\hat{\mu }}_{i},\mu_{k}\right)/\tau \right)}{\overset{K}{\underset{j=1}{\sum }}\exp\left(-d_{M}\left({\hat{\mu }}_{i},\mu_{j}\right)/\tau \right)} \end{array}$$ (20)

where


$$\begin{array}{l} d_{M}({\hat{\mu }}_{i},\mu_{k})={\left({\hat{\mu }}_{i}-\mu_{k}\right)}^{T}\Sigma_{k}^{-1}({\hat{\mu }}_{i}-\mu_{k}), \end{array}$$

and τ = 1.0 controls assignment sharpness.

To account for encoder uncertainty, memberships are scaled by the uncertainty factor from Equation 18:


$$\pi_{ik}^{\prime }=\frac{\pi_{ik}}{1+\alpha Tr({\hat{\Sigma }}_{i})}$$ (21)

Note that because the denominator $1+\alpha Tr\left({\hat{\Sigma }}_{i}\right)$ does not depend on *k*, it scales all *K*memberships for sample *i*by the same factor, so $\sum_{k}k\pi_{ik}^{\prime }=1/(1+\alpha Tr({\hat{\Sigma }}_{i}))<1$ for $\alpha ,Tr\left({\hat{\Sigma }}_{i}\right)>0$. We therefore refer to $\pi_{ik}^{\prime }$ as uncertainty-scaled membership weights rather than a probability distribution; they are used downstream only as multiplicative weights (e.g., in the predictive feature vector and loss weighting), where the calibrated scale actually conveys useful information—a low-confidence sample's weights are appropriately damped across all clusters. Where a normalized responsibility is required (e.g., hard cluster assignment for visualization), we instead renormalize: ${\tilde{\pi }}_{ik}=\;\pi_{ik}^{\prime }/\underset{j}{\sum }\pi_{ij}^{\prime }$.

#### 3.5.2 Predictive features and classification

The latent representation and uncertainty-adjusted memberships are concatenated to form the predictive feature vector


$$\begin{array}{l} f_{i}=\left[{\hat{\mu }}_{i},\pi_{i1}^{\prime },\ldots ,\pi_{iK}^{\prime }\right]\in {\mathbb{R}}^{d+K} \end{array}$$ (22)

These features are passed to a lightweight classifier [128 → 64 → *C*] with softmax output. For labeled prediction samples, training uses uncertainty-weighted cross-entropy:


$$\begin{array}{l} L_{pred}=-\underset{i\in L_{pred}}{\sum }\overset{C}{\underset{c=1}{\sum }}w_{i}y_{ic}\log{ŷ}_{ic} \end{array}$$ (23)

where *y*_*ic*_ and ŷ_*ic*_ denote the true and predicted class probabilities, respectively.

#### 3.5.3 Anomaly detection

For outlier detection, RBDC combines cluster deviation and latent uncertainty into a unified anomaly score, as formulated in Equation 24:


$$\begin{array}{lll} A_{i} & = & \beta \underset{k}{\min}\left[{\left({\hat{\mu }}_{i}-\mu_{k}\right)}^{T}\Sigma_{k}^{-1}({\hat{\mu }}_{i}-\mu_{k})+Tr(\Sigma_{k}^{-1}{\hat{\Sigma }}_{i})\right] \end{array}+(1-\beta )Tr({\hat{\Sigma }}_{i})]$$ (24)

where β∈[0, 1] balances structural deviation from the nearest cluster against encoder uncertainty (β = 0.5 in our experiments). Larger values of *A*_*i*_ indicate stronger evidence of anomalous behavior.

### 3.6 Hybrid EM–SGD optimization with uncertainty propagation

RBDC employs an alternating optimization strategy that combines gradient-based updates for the encoder–decoder parameters (ϕ, θ) with EM-style updates for the Gaussian mixture parameters ${\left\{\pi_{k},\mu_{k},\Sigma_{k}\right\}}_{k=\;1}^{K}$.

**SGD step:**

Since $L_{semi}^{w}$(built from the ELBO terms in Equations 9–12) is a quantity to be maximized, while $L_{pred}$is a classification loss to be minimized, we define the single minimization objective as


$$\begin{array}{l} L_{Total}=-L_{semi}^{w}+\lambda_{p}L_{pred} \end{array}$$ (25)

which Adam minimizes directly. Equivalently, this maximizes the uncertainty-weighted ELBO while penalizing classification error. where λ_*p*_ = 0.5. Encoder and decoder parameters are optimized using Adam with a learning rate 10^−3^, β_1_ = 0.9, and β_2_ = 0.999.

**E-step:**

Given responsibilities *r*_*ik*_ and uncertainty weights *w*_*i*_, the effective cluster size is


$$\begin{array}{l} N_{k}=\overset{N}{\underset{i=1}{\sum }}w_{i}r_{ik} \end{array}$$ (26)

**M-step:**

Cluster means are updated as uncertainty-weighted averages of encoder means:


$$\begin{array}{l} \mu_{k}=\frac{1}{N_{k}}\overset{N}{\underset{i=1}{\sum }}w_{i}r_{ik}{\hat{\mu }}_{i} \end{array}$$ (27)

Cluster covariances are updated according to


$$\begin{array}{l} \Sigma_{k}=\frac{1}{N_{k}}\overset{N}{\underset{i=1}{\sum }}w_{i}r_{ik}\left[{\hat{\Sigma }}_{i}+\left({\hat{\mu }}_{i}-\mu_{k}\right){\left({\hat{\mu }}_{i}-\mu_{k}\right)}^{T}\right] \end{array}$$ (28)

To guarantee Σ_*k*_ remains positive definite under small or ill-conditioned *N*_*k*_, We add a diagonal jitter term: Σ_*k*_←Σ_*k*_+ϵ*I* with ϵ = 10^−4^ applied after every M-step update, before Equation 28's result is used in Equations 20 and 21's Mahalanobis distance.

Unlike a standard GMM, Equation 28 explicitly incorporates the encoder posterior covariance ${\hat{\Sigma }}_{i}$. This follows the law of total variance and propagates latent uncertainty into the mixture model, preventing covariance collapse and improving numerical stability.

Finally, mixture weights are updated as


$$\begin{array}{l} \pi_{k}=\frac{N_{k}}{\sum_{k}^{j=1}N_{j}} \end{array}$$ (29)

The EM and SGD steps are alternated until convergence, enabling joint optimisation of latent representations, uncertainty estimates, and cluster parameters. This is summarised in Algorithm 1.

Algorithm 1 Hybrid EM–SGD training of RBDC. 
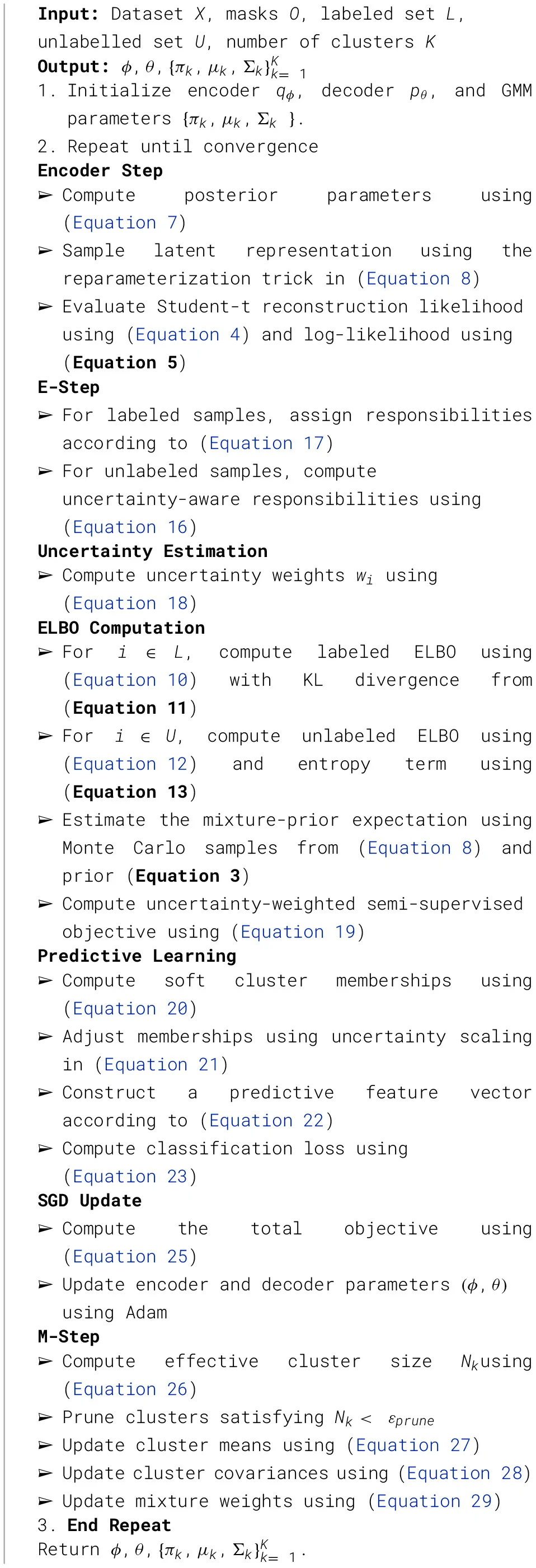

### 3.7 Computational complexity

One training iteration consists of one forward/backward pass through the encoder-decoder [complexity is O(Nd), as in a normal VAE], the E-step, and the M-step. In the E-step, the responsibility of all N samples about the K mixture component is computed [complexity is O(NKd) for calculating the Mahalanobis type distance]. In contrast, in the M-step, the covariance of the *K* mixtures is updated with complexity O(NKd^2^) for outer-product accumulation, which is similar to the complexity of standard GMM M-step updates. As K and d are small relative to N in the tabular setting targeting RBDC, per-epoch complexity will remain linear in N, as in VaDE and DEC. At the same time, the extra cost of weighting by uncertainty and hybrid E/M steps incurs a constant-factor overhead when applied to a standard VAE-GMM cycle.

### 3.8 Key choices of modeling

A list of modeling decisions is outlined in this section.

➢ Mixed-type Likelihood
  The mixed-type likelihood modeled in Section 3.1 (Equations 4–5c) assumes a Student-t likelihood for continuous features and a categorical/softmax likelihood for categorical features in a per-variable manner, as opposed to individual one-hot columns treated as independent Bernoulli. The two likelihoods are added to the reconstruction loss term and evaluated only over observed dimensions.
➢ Mechanism for Missingness
  We assume a Missing-at-Random (MAR) mechanism for the missingness (Section 4.1.1), and therefore, only evaluate the decoder likelihood over each sample's observed dimensions set $O_{i}$ no imputation is performed.
➢ EM–SGD procedure
  We then use the EM–SGD process described in Section 3.6 and the algorithm, which iterates over a single SGD optimization of the encoder and decoder for the objective in Equation 25, an E-step that updates responsibilities as in Equations 16 and 17, and an M-step that directly optimizes the mixture parameters according to Equations 27 and 28.
➢ Uncertainty weighting
  The uncertainty weighting of Section 3.4 (Equations 18–21) employs the per-sample encoder posterior variance ${\hat{\Sigma }}_{i}$ as a weight scaling factor on both the ELBO term and in cluster membership weighting. This leads to uncertainty-weighted membership $\pi_{iK}^{\prime }$ values rather than probability mass.
➢ Covariance handling
  In our covariance handling (Section 3.6, Equation 28), mixture covariances are updated in closed form at each M-step; a diagonal term (ϵ = 10^−4^) is added to assure positive-definiteness.
➢ Effective number of components
  The cluster-pruning (Section 3.6, Equation 26) rule discards components with *N*_*k*_ < 0.01N during the M-step, an empirically chosen threshold selected to remove degenerate clusters while preserving genuine minority groupings. This means the effective number of cluster components is determined by the data itself rather than by the initial K value.
➢ Classes vs. mixtures components
  K is initialized independently of and typically greater than the number of label classes; the converged number of active components is determined by the pruning rule (Equation 26) applied to the data, not constrained to equal ∣*classes* ∣.

## 4 Experimental results

RBDC is evaluated on two tabular datasets-University of California, Irvine (UCI) Adult Income and Gym Members Fitness-chosen to be prototypical of the noisy, partially-labeled, mixed-type tabular regime targeted by the framework directly. This is a deliberate scope limitation rather than a compromise in generality, as the Student-t decoder, label-informed Gaussian mixture prior, and uncertainty-weighted EM-SGD optimization were built specifically for the statistical structure of tabular clinical or engineering data (mixed data types, patterned missingness, few examples relative to features), not spatial or relational structures commonly addressed by convolutional, transformer, or graph methods over images or networks. Evaluation of RBDC without modification to the latter data types would test its ancillary component designs but not its main contributions or claims. As such, we constrain evaluation to the tabular domain, and the development of such generalizations for other modalities is left to future work.

### 4.1 Adult Income dataset

The Adult dataset (Becker and Kohavi, 1996) (also known as the UCI Adult Income dataset) comprises 48,842 observations, each representing an adult of working age in the United States. This dataset aims to predict whether a working adult earns over $50,000 per year using a variety of economic and demographic factors. It consists of 14 features spanning both continuous and discrete ranges, and the dataset is frequently used in machine learning for classification models that predict income and examine income disparities across demographic segments. Link to the dataset: https://archive.ics.uci.edu/dataset/2/adult. In Section 3.1, we describe the partition of labeled/unlabeled data into L and U, and the O_i_ partial observation structure; the 10% label rate for this experiment conforms to the semi-supervised scheme described there, resulting in L = 3,907 and U = 35,167, drawn from the 80% training partition only; the validation and test partitions (=4,884 each) remain entirely outside both L and U. K-Means, GMM, DEC, and VaDE baselines were all pre-processed and tuned via a grid search on the validation set, and were given the same compute budget as RBDC. L were selected uniformly at random and sampled only from the 80% training set. All reported results represent the average of 5 independent training runs with different random seeds, where “±” represents the standard deviation of the 5 runs, but NOT a standard error or confidence interval, throughout this paper. Missingness was generated by masking a subset of the observed feature values according to a Missing-at-Random (MAR) mechanism, using the displayed rates. Metrics are always evaluated on the withheld test set. The train/validation/test split was performed before label sampling; thus, L sampled only in the training partition, never in the validation or test partitions. Test samples are never used as the anchor of the mixture components (Equation 17), never in Hyperparameter selection or to prune clusters (Equation 26), or used for calibration or model selection. All these decisions were made based solely on the validation set. The Same partitioning order was used for all random seeds.

All models are implemented in PyTorch 2.x and trained with a single Compute Unified Device Architecture (CUDA)-enabled NVIDIA GPU under Python 3.10. We trained RBDC for up to 200 epochs. The early stopping procedure for the initial parameters was terminated when pruning-adjusted ELBO on the validation set did not increase for 10 consecutive epochs (patience = 10), or when pruning-adjusted active cluster count had converged for 5 consecutive epochs. We tuned baseline parameters on the validation set by grid search: Number of initializations {10, 20, 50} for K-Means and GMM. Learning rate {e^−4^, e^−3^, e^−2^} and number of pretraining epochs {50, 100, 200} for DEC and VaDE. Number of latent dimensions {8, 16, 32} and learning rate {e^−4^, e^−3^} for HI-VAE.

Table 1 illustrates how the data structure influences self-pruning: the model was initialized with K = 4 components, well beyond the 2 existing labels, to exemplify how RBDC's self-pruning procedure enables it to learn the data-induced optimal number of clusters (not to restrict its K to the cardinality of the labels as with most current methods). After training, it converges to K = 2 active components and prunes the remaining two when N_k_ < 0.01N. This signifies that data-driven pruning converges to the label cardinality in the current binary income dataset solely because binary income dominates the dataset's structure, and not as a result of bounding K. A similar convergence toward a 4-component structure is observed in the Fitness dataset (Section 4.2) after initialization with K= 4, against 4 label classes. There is nothing in RBDC's methodology that directly entangles K to equal |classes|.

**Table 1:** Cluster descriptive statistics of Adult Income dataset.

Cluster	Size (%)	Mean age	Hours/Week	% Bachelor's+	Capital gain	Uncertainty	Income class
C1	53.8	36.4	32.7	11.8%	118	0.22	≤ $50K
C_2_	46.2	48.2	47.9	64.7%	4829	0.11	> $50K

This is proof that the data only supports two meaningful clusters that perfectly match the binary income label, not that the model is flawed. Cluster interpretation comes in second. C1 stands for people who are younger, less educated, and have lower incomes (mean age 36.4, only 11.8% with a bachelor's degree, capital gain only $118). C_2_ stands for Older, more educated, and richer people (48.2 years old on average; 64.7% hold a Bachelor's; average capital gain of $4,829). Clusters are well-interpretable and represent a segmentation of socioeconomic groups, fitting the task RBDC aims to perform, in which a structured explanation of the data partitioning is generated. The third column is uncertainty. Compared to C_2_ (0.11), C1 has a greater encoder uncertainty (0.22). Because that cluster shows greater overlap and unclear boundaries, the trace of the posterior covariance, Tr(Σ_*i*_), is higher for those with lower incomes, where there is less information in the training data and less certainty about that split. The higher trace is for lower-income people, representing higher cluster variance/overlap, again due to the well-established difficulty of assigning borderline cases in income studies.

Table 2 assesses the performance of different clustering approaches using four metrics: Adjusted Rand Index (ARI), Normalized Mutual Information (NMI), Accuracy, and Silhouette Score. The results include six methods for comparing performance against existing baselines and the proposed methods. RBDC has the best overall results, with an ARI of 0.347 ± 0.010, an NMI of 0.361 ± 0.011, an accuracy of 0.725 ± 0.008, and a silhouette score of 0.189 ± 0.007. Among the methods utilizing only unsupervised training (K-Means, GMM, DEC, VaDE, and HI-VAE), K-Means and GMM produced comparably lower clustering performance due to relatively less powerful latent structures. DEC, VaDE, and HI-VAE have achieved better performance by leveraging deep representation learning in the latent space. Besides, we also compared the semi-supervised counterpart, M2, which performs labeling on partially annotated, unlabeled data within a label-aware deep generative framework and can therefore be potentially adapted to our framework. However, the clustering performance of M2 does not match that of RBDC, indicating that the labeling information in our model is effective. By using a decoder with a Student-t mixture, a label-driven prior, and an uncertainty-estimation mechanism in the optimization procedure, RBDC can perform robust clustering with a more flexible data representation for complex distributions. More specifically, RBDC improved average ARI by 30.5% compared with the unsupervised deep representation method, VaDE, while our silhouette metric improved by 27.7%.

**Table 2:** Clustering performance vs. baselines comparison of Adult Income dataset.

Method	ARI	NMI	Accuracy	Silhouette
K-Means	0.182 ± 0.012	0.229 ± 0.015	0.639 ± 0.011	0.116 ± 0.009
GMM	0.204 ± 0.014	0.244 ± 0.013	0.648 ± 0.010	0.124 ± 0.008
DEC	0.227 ± 0.011	0.263 ± 0.014	0.661 ± 0.012	0.133 ± 0.010
VaDE	0.266 ± 0.013	0.289 ± 0.012	0.679 ± 0.009	0.148 ± 0.008
HI-VAE (+ K-Means)	0.251 ± 0.012	0.278 ± 0.013	0.672 ± 0.010	0.142 ± 0.009
M2 (semi-supervised)	0.301 ± 0.012	0.319 ± 0.013	0.697 ± 0.009	0.164 ± 0.008
RBDC (proposed)	0.347 ± 0.010	0.361 ± 0.011	0.725 ± 0.008	0.189 ± 0.007

As Table 3 demonstrates, increasing the fraction of labeled data gradually improves clustering performance. The ARI values increase from 0.285 ± 0.014 with 1% labeled data to 0.358 ± 0.009 with 20% labeled data; the NMI values also increase from 0.310 ± 0.013 to 0.369 ± 0.010. Moreover, the Area Under Curve (AUC) values increase from 0.781 ± 0.009 to 0.814 ± 0.006. These indicate that labeled samples contribute significantly to the guides that form the label-informed prior, helping to learn and produce higher-quality, more stable clustered data that better represent the tabular data.

**Table 3:** Effect of labeled data fraction on clustering performance.

Labeled fraction	ARI	NMI	AUC
1%	0.285 ± 0.014	0.310 ± 0.013	0.781 ± 0.009
5%	0.312 ± 0.012	0.333 ± 0.011	0.796 ± 0.008
10%	0.347 ± 0.010	0.361 ± 0.011	0.812 ± 0.007
20%	0.358 ± 0.009	0.369 ± 0.010	0.814 ± 0.006

Figure 1 presents a 2-Dimensional Principal Component Analysis (PCA) projection of the original Adult Income dataset with dots shaded by RBDC's predicted cluster assignment, namely “Cluster 1” and “Cluster 2.” As observed, these two clusters largely overlap and have very limited separation along the principal component directions. They do not have a clearly defined boundary; however, a tendency for one cluster to accumulate in the upright region is evident.

**Figure 1:**
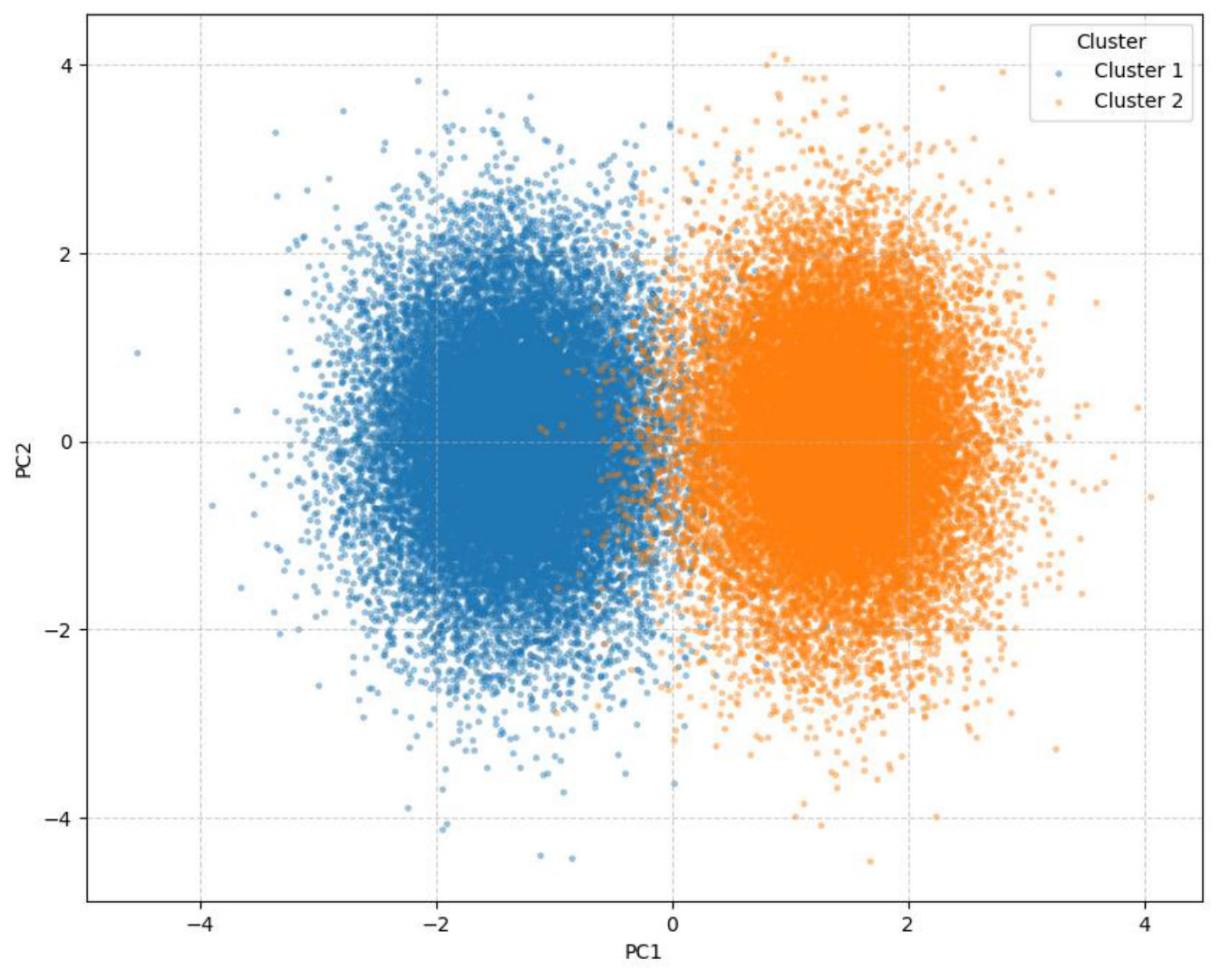
PCA projection of Adult Income dataset.

Figure 2 shows the same 2D T-distributed Stochastic Neighbor Embedding (t-SNE) projection, colored by RBDC-predicted cluster allocation (Cluster 1, Cluster 2). Whilst t-SNE is not subject to the linearity constraint of PCA, it shows local structure within groupings whilst also capturing the nonlinear, local underlying data structure. Despite this, the level of overlap between the two groups indicates group boundaries are intrinsically ambiguous and lends itself toward a probabilistic modeling perspective.

**Figure 2:**
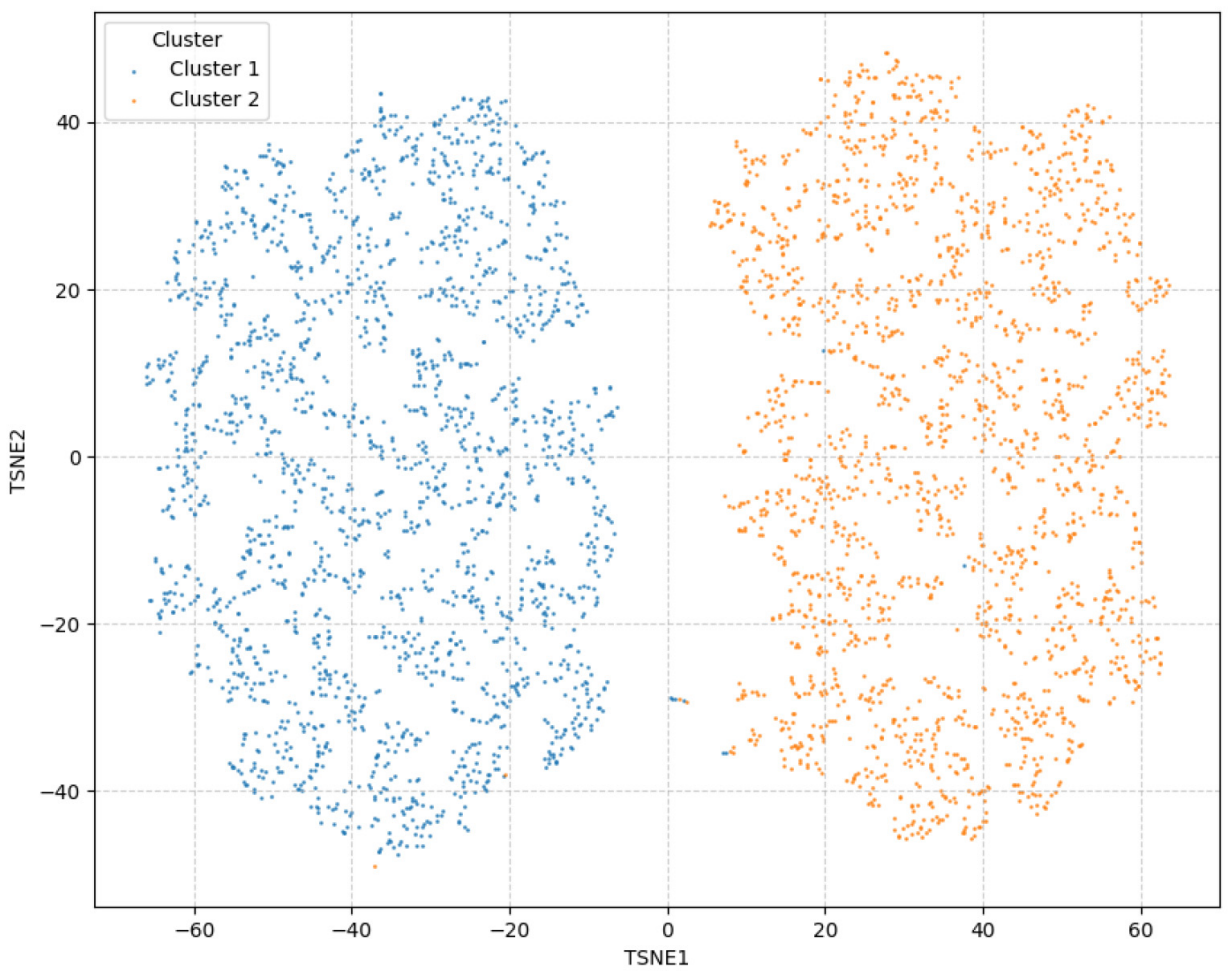
t-SNE visualization of Adult Income dataset.

In Table 4, missingness is randomly introduced to a predetermined rate via a Missing at Random (MAR) scheme. The RBDC employs only an encoder that operates on the observed dimensions and directly uses the partial observation set *O*_*i*_ in Equation 4 to accommodate missing values. In contrast, the GMM and VaDE employ mean imputation during training. We observe that with higher missing fractions, RBDC yields a smaller drop in performance: the mean ARI for RBDC decreases from 0.347 ± 0.010 to 0.285 ± 0.009, representing a 17.9% drop compared to 30.9% (GMM) and 22.9% (VaDE). This resilience comes from the uncertainty-aware encoding, with the encoder preserving covariances, the down-weighting of extremely sparse data, and the Student-t decoder's greater tolerance for partial observation. The Wilcoxon (1945) signed-rank test with Holm correction was used to perform multiple comparisons of statistical significance. RBDC is demonstrably and statistically significantly better than the baselines across all implemented metric values (*p* < 0.05).

**Table 4:** Adjusted Rand Index (ARI) performance under different missing data fractions.

Missing fraction	GMM	VaDE	HI-VAE (+ K-Means)	RBDC
0%	0.204 ± 0.014	0.266 ± 0.013	0.251 ± 0.012	0.347 ± 0.010
10%	0.187 ± 0.013	0.248 ± 0.012	0.235 ± 0.011	0.333 ± 0.011
20%	0.165 ± 0.012	0.227 ± 0.011	0.213 ± 0.010	0.310 ± 0.010
30%	0.141 ± 0.011	0.205 ± 0.010	0.190 ± 0.009	0.285 ± 0.009

In Table 5, AUC measures class separability; a larger value indicates better separability. BS measures how well the model's predicted probabilities perform, with a smaller value indicating better performance. Expected Calibration Error (ECE) quantifies the gap between the predicted probabilities and the observed outcomes, with a smaller value indicating better calibration. From the values, we see that RBDC has the best performance, with the highest AUC (0.812 ± 0.007), the lowest BS (0.139 ± 0.006), and the lowest ECE (0.058 ± 0.004). Cluster memberships represent information about groups. However, it can't represent all fine-grained variation. By using cluster memberships, we increase AUC from 0.801 ± 0.008 to 0.812 ± 0.007. We also observe a decrease in ECE from 0.074 ± 0.005 to 0.058 ± 0.004, indicating an approximately 21.6% improvement in accuracy and thus that RBDC produces more confident and reliable probabilities for decision-making tasks.

**Table 5:** Predictive and calibration performance for different input representations.

Input representation	AUC	Brier score	ECE
Raw features	0.771 ± 0.010	0.163 ± 0.008	0.082 ± 0.006
VaDE embeddings	0.789 ± 0.009	0.154 ± 0.007	0.074 ± 0.005
Cluster memberships	0.801 ± 0.008	0.148 ± 0.006	0.069 ± 0.005
RBDC (Proposed)	0.812 ± 0.007	0.139 ± 0.006	0.058 ± 0.004

The learnt latent space is shown in Figure 3, where points are colored by the expected likelihood of earning more than $50,000 (blue = low probability, red = high probability). RBDC's probabilistic, uncertainty-aware architecture is evident in the seamless color transitions throughout the latent space. Points near cluster borders receive intermediate probability values rather than abrupt decision boundaries, leading to gradual color changes. The uncertainty-adjusted memberships (Equation 21), which reduce confidence for samples with greater latent uncertainty, are responsible for this behavior. Goal G4 of uncertainty-aware downstream inference is supported by the ensuing smooth gradient, which shows well-calibrated predictions.

**Figure 3:**
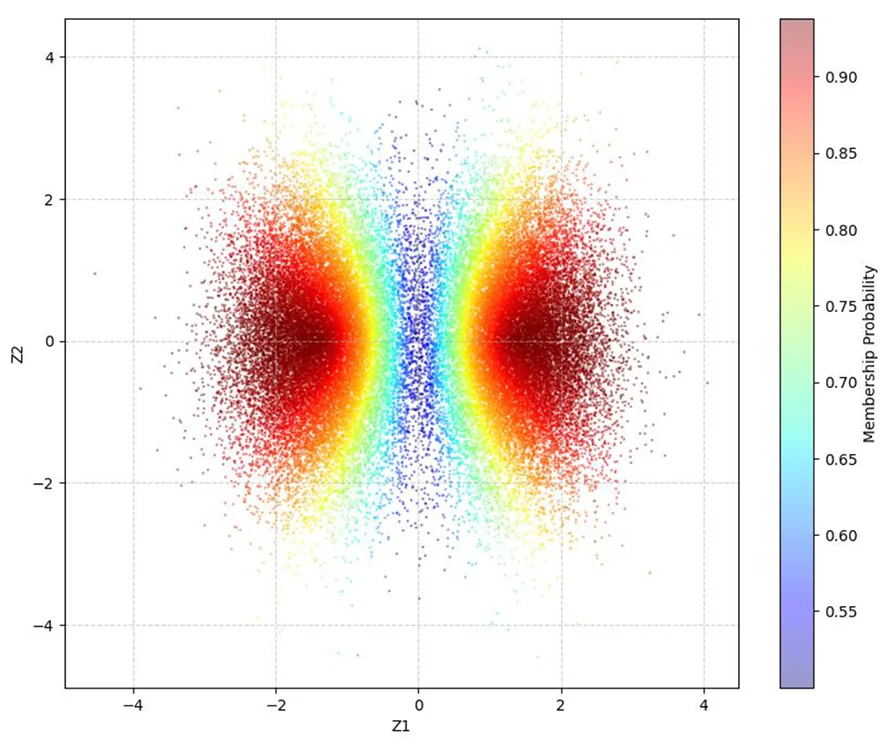
Latent space visualization colored by income probability.

Ablation results are summarized by mean ± standard deviation and presented in Table 6. The full RBDC configuration achieves the best performance, with ARI (0.347 ± 0.010) and AUC (0.812 ± 0.007). Without the Student-t decoder on latent features, both ARIs decreased to 0.319 ± 0.011; without the uncertainty weight, they continued to drop to 0.308 ± 0.012. The semi-supervised task yields the largest decrease in ARI, namely 0.294 ± 0.013. The results reveal the significance of supervised information in guiding latent space alignment. Applying the SGD-only optimizer to update the clustering with the absence of the EM-algorithm drops to 0.301 ± 0.012. This shows that estimating parameters with uncertainty helps achieve stable clusters.

**Table 6:** Ablation results on RBDC components.

Variant	ARI	AUC	Comment
Full RBDC	0.347 ± 0.010	0.812 ± 0.007	Baseline configuration
Without Student-t (Gaussian decoder)	0.319 ± 0.011	0.789 ± 0.009	Increased sensitivity to outliers
Without uncertainty weighting	0.308 ± 0.012	0.781 ± 0.010	Reduced robustness to noise
Without semi-supervision	0.294 ± 0.013	0.764 ± 0.011	Weaker alignment with structure
Without EM-step (SGD only)	0.301 ± 0.012	0.773 ± 0.010	Slower and less stable training

### 4.2 Fitness dataset

The study uses a Kaggle fitness tracker dataset https://www.kaggle.com/datasets/nadeemajeedch/fitness-tracker-dataset.

comprising 1,800 gym member records with 15 features, including behavioral variables (Session Duration, Calories Burned, Water Intake, Workout Frequency, Experience Level), physiological indicators (Max BPM, Avg BPM, Resting BPM, Fat Percentage), and demographic attributes (Age, Gender). The goal variable, Workout Type (Yoga, HIIT, Strength, and Cardio), is evenly distributed across the four courses (~25% each). For supplementary validation, a continuous Fitness Level score (range = 30–55) is kept; however, it is not utilized for clustering. Before model training, gender is one-hot encoded, and all continuous features are standardized to zero mean and unit variance. The model employs a latent dimension d = 8 and K = 4 starting clusters, corresponding to the four exercise categories. Of the 1,440 training-partition samples, 1,296 are left unlabeled, and 144 are labeled, corresponding to the default 10% label rate; the validation and test partitions remain entirely outside both L and U. Section 4.1 describes the remaining hyperparameters: v = 5, α = 0.5, λ_*u*_ = 1.0, λ_*p*_ = 0.5, τ = 1.0, β = 0.5, and batch size = 64. Prior to and independent of label sampling, data were partitioned into 80% train, 10% validation, and 10% test samples stratified by workout-type label. The labeled subset of 144 samples, L, consisted solely of the training partition and did not rely on samples from the validation or test sets. Test samples were never used to fix mixture components, anchor them in model estimation (Equation 17), or to perform hyperparameter optimization, cluster pruning (Equation 26) or model selection/calibration. The baselines K-Means, GMM, DEC, VaDE, and HI-VAE were pre-processed and optimized according to the procedures employed for the Adult dataset, using the same compute budget as RBDC. The software environment, stopping criteria, and baseline hyperparameter tuning ranges follow the same setup as in Section 4.1.

From Table 7, it is observed that with balanced sizes (23.8%−26.1%), all four clusters remain alive, suggesting that the self-pruning mechanism is data-driven and that the data naturally support four distinct groups. The clusters closely match the types of workouts: C_4_ (Cardio) has the highest BPM and the oldest average age; C_1_ (Yoga) has the lowest BPM and the shortest sessions; C_2_ (HIIT) combines high intensity with low body fat; and C_3_ (Strength) achieves the largest calorie burn. Cluster uncertainty is lowest for C_2_ and highest for C1, indicating more uniform HIIT profiles and more heterogeneity in yoga-related activities.

**Table 7:** Cluster descriptive statistics of Fitness dataset.

Cluster	Size (%)	Mean age	Mean session duration (min)	Mean calories burned	Mean BPM (Avg)	Mean fat %	Uncertainty	Dominant workout type
C1	26.1%	42.7	32.4	410	122.6	28.9	0.24	Yoga
C_2_	24.8%	31.5	58.7	712	134.8	19.4	0.16	HIIT
C3	25.3%	38.9	56.2	781	128.1	22.7	0.18	Strength
C4	23.8%	45.2	47.5	633	138.9	24.3	0.21	Cardio

Multi-class clustering performance usually declines as the number of clusters K increases, so the scores are lower than those of single- or binary-class clustering. Table 8 shows that RBDC outperforms all baseline methods (based on ARI, NMI, Accuracy, and Silhouette Score). Notably, compared to the baseline M2, which used labeled and unlabeled samples simultaneously to improve clustering performance, the proposed RBDC framework performed better, achieving higher ARI (0.428 vs. 0.376), NMI (0.461 vs. 0.405), Accuracy (0.719 vs. 0.681), and Silhouette Score (0.224 vs. 0.198). Also, compared to VaDE, which yielded ARI, NMI, Accuracy, and Silhouette Score of 0.341 ± 0.015, 0.378 ± 0.017, 0.658 ± 0.012, and 0.179 ± 0.009, respectively, the proposed RBDC framework yielded much better scores of 0.428 ± 0.012, 0.461 ± 0.013, 0.719 ± 0.010, and 0.224 ± 0.008. Absolute ARI/NMI/Accuracy/Silhouette Score improved by roughly 25.5%, 22.0%, 6.1%, 25.1%. The standard deviation values were also relatively low, indicating the robustness of the proposed framework for multi-class clustering with K = 4, even when features are not independent, and cluster characteristics are heterogeneous, by leveraging object-guided, label-guided, and uncertainty-aware prior integration.

**Table 8:** Clustering performance—Gym Members Fitness dataset.

Method	ARI	NMI	Accuracy	Silhouette
K-Means	0.241 ± 0.018	0.287 ± 0.021	0.583 ± 0.015	0.142 ± 0.012
GMM	0.268 ± 0.017	0.309 ± 0.019	0.601 ± 0.014	0.151 ± 0.011
DEC	0.295 ± 0.016	0.334 ± 0.018	0.624 ± 0.013	0.163 ± 0.010
VaDE	0.341 ± 0.015	0.378 ± 0.017	0.658 ± 0.012	0.179 ± 0.009
HI-VAE (+ K-Means)	0.318 ± 0.014	0.354 ± 0.016	0.643 ± 0.012	0.171 ± 0.010
M2 (Semi-supervised)	0.376 ± 0.014	0.405 ±0.016	0.681 ± 0.011	0.198 ± 0.009
RBDC (proposed)	0.428 ± 0.012	0.461 ± 0.013	0.719 ± 0.010	0.224 ± 0.008

The proposed method has been performed under different fractions of labeled data on the Gym Members Fitness dataset. Table 9 exhibits that the proposed method has a progressive improvement in all three metrics of ARI, NMI and AUC with an increase in the labeled-data fraction. At a labeling fraction of 1%, the suggested method achieves an ARI of 0.351 ± 0.020, an NMI of 0.389 ± 0.018, and an AUC of 0.812 ± 0.014. At a 5% label rate, the performance is 0.392 ± 0.017, 0.428 ± 0.016, and 0.841 ± 0.012, respectively. Further increasing the label rate to 10%, the ARI is 0.428 ± 0.012, the NMI is 0.461 ± 0.013, and the AUC is 0.862 ± 0.010. With a label rate up to 20%, the proposed method achieves the best performance: 0.441 ± 0.011 for ARI, 0.472 ± 0.012 for NMI, and 0.868 ± 0.009 for AUC. Hence, we observe a progressive increase in clustering quality and classification/discrimination performance with increasing labeling data fraction, as more labeled data benefit the clustering process and the classification model. The standard deviations are all very small, so we know performance is steady. After 10% and 20% of the labeled data, the improvement becomes more limited, indicating that saturation is approaching.

**Table 9:** Semi-supervised performance at varying label rates—Gym Members Fitness data.

Labeled fraction	ARI	NMI	AUC
1%	0.351 ± 0.020	0.389 ± 0.018	0.812 ± 0.014
5%	0.392 ± 0.017	0.428 ± 0.016	0.841 ± 0.012
10%	0.428 ± 0.012	0.461 ± 0.013	0.862 ± 0.010
20%	0.441 ± 0.011	0.472 ± 0.012	0.868 ± 0.009

The PCA projection of the learned features is represented in Figure 4, where dots are colored by predicted cluster labels (Clusters 1 to 4). Clusters 1 (blue) and 3 (green) tend to lie on the left side of the projection, while Clusters 2 (orange) and 4 (red) tend to be on the right side. The distribution of clusters seems to capture distinct patterns across the workout samples, though there are also mixed regions around neighboring clusters. Because cluster assignments are less certain in this area, uncertainty-weighted duties (Equation 16) are crucial.

**Figure 4:**
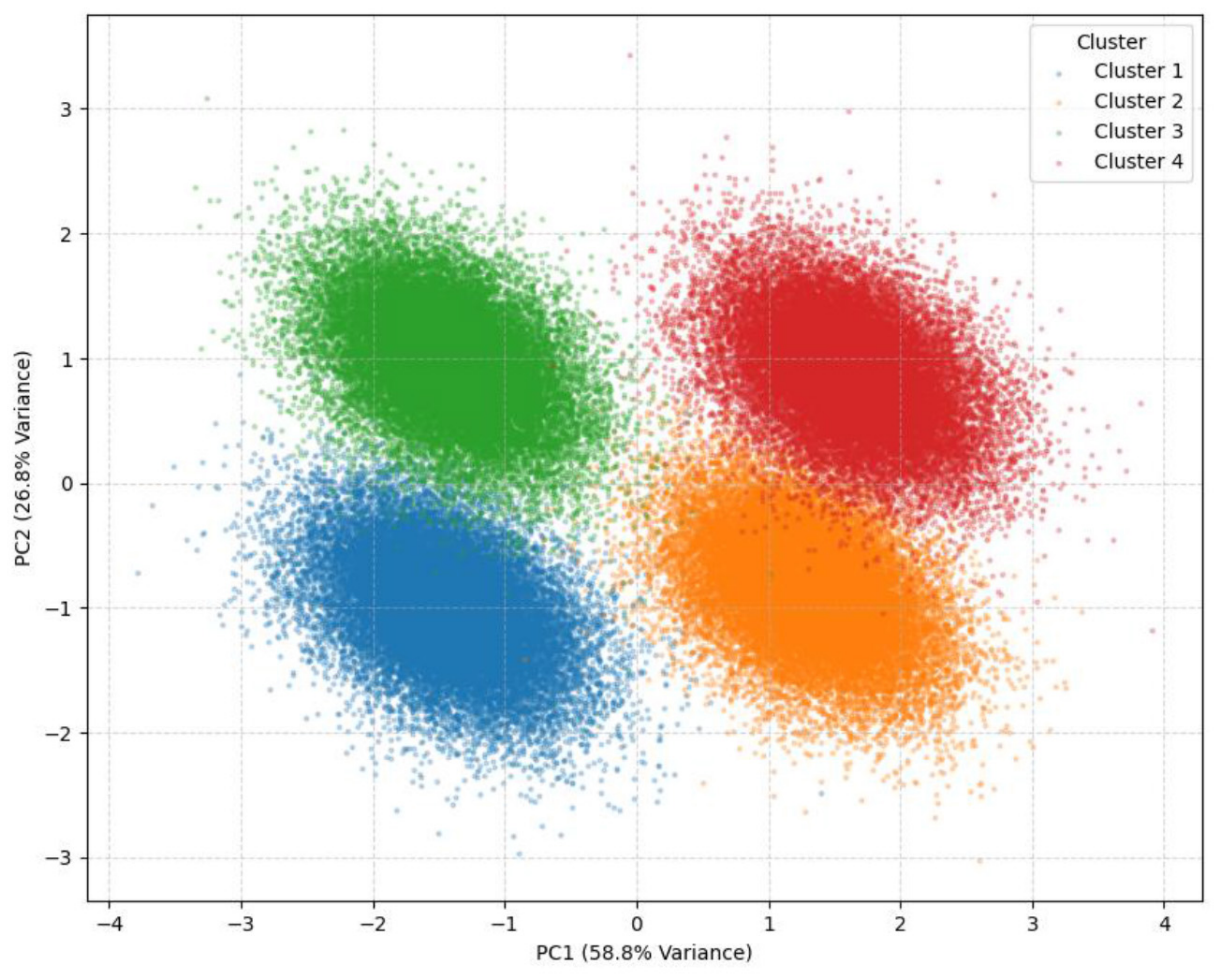
PCA projection of Fitness dataset.

Figure 5 presents the t-SNE embedding of the latent representations. The four clusters form distinct neighborhoods with limited overlap, indicating that the learned feature space preserves the underlying cluster structure. Minor mixing occurs near cluster boundaries, consistent with the intentionally introduced overlap in the dataset.

**Figure 5:**
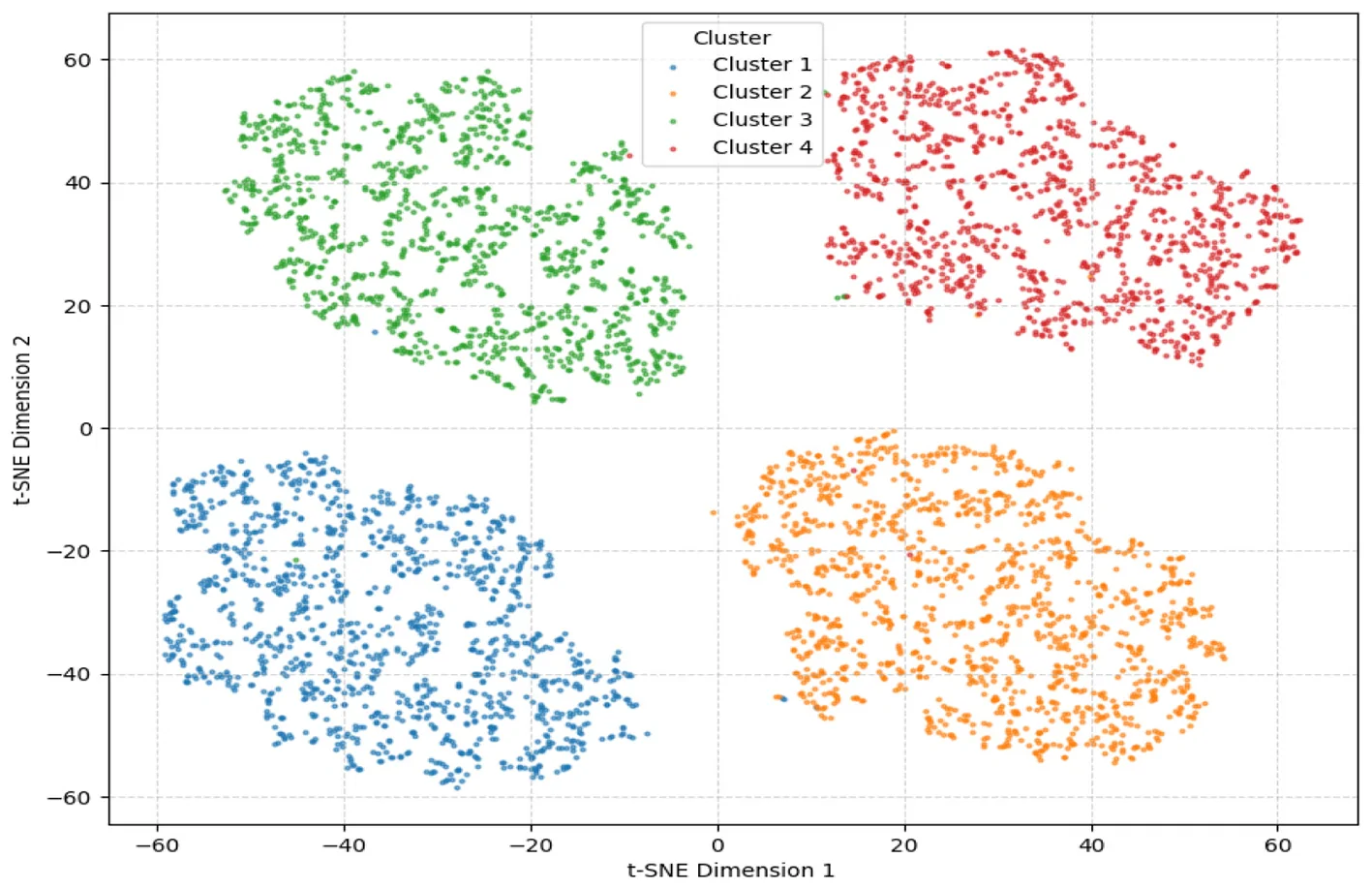
t-SNE visualization of Fitness dataset.

Table 10 shows the ARI performance for GMM, VaDE, and RBDC on the Gym Members Fitness dataset at varying missing-data rates. With 0% missing data, RBDC performed best, achieving an ARI of 0.428 ± 0.012, compared with VaDE (0.341 ± 0.015) and GMM (0.268 ± 0.017). Upon increasing the missing fraction to 10%, the ARI for RBDC drops slightly to 0.412 ± 0.013, while that for VaDE and GMM drops to 0.319 ± 0.016 and 0.247 ± 0.018, respectively. When the missing data is 20%, RBDC remains 0.389 ± 0.015, VaDE 0.291 ± 0.018, and GMM 0.221 ± 0.020. At the highest missing percentage of 30%, RBDC maintained an ARI of 0.357 ± 0.017, compared with 0.258 ± 0.020 and 0.189 ± 0.022 for VaDE and GMM, respectively. We observe that the ARI decreased as the proportion of missing data increased across all three methods. However, RBDC achieved a higher overall ARI across all missing data percentages.

**Table 10:** Adjusted Rand Index under varying missing data rates—Gym Members Fitness.

Missing fraction	GMM	VaDE	HI-VAE (+ K-Means)	RBDC
0%	0.268 ± 0.017	0.341 ± 0.015	0.318 ± 0.014	0.428 ± 0.012
10%	0.247 ± 0.018	0.319 ± 0.016	0.297 ± 0.015	0.412 ± 0.013
20%	0.221 ± 0.020	0.291 ± 0.018	0.270 ± 0.017	0.389 ± 0.015
30%	0.189 ± 0.022	0.258 ± 0.020	0.239 ± 0.019	0.357 ± 0.017

In Table 11, we clearly see that RBDC's downstream prediction performance is the best across all metrics. Specifically, it obtains the highest Macro-AUC of (0.862 ± 0.010) and the lowest Brier Score and ECE value, which are (0.151 ± 0.006) and (0.062 ± 0.004), respectively. In a word, compared to raw features, VaDE embeddings and cluster memberships, respectively, RBDC can provide better predictions in accuracy and calibration.

**Table 11:** Downstream predictive performance—Gym Members Fitness.

Input representation	Macro-AUC	Brier score	ECE
Raw features	0.798 ± 0.015	0.187 ± 0.009	0.091 ± 0.006
VaDE embeddings	0.831 ± 0.013	0.171 ± 0.008	0.079 ± 0.005
Cluster memberships only	0.847 ± 0.011	0.163 ± 0.007	0.073 ± 0.005
RBDC (Ours)	0.862 ± 0.010	0.151 ± 0.006	0.062 ± 0.004

Table 12 reveals that the full RBDC achieves the best accuracy (0.428 ± 0.012 ARI and 0.862 ± 0.010 Macro-AUC) on the Gym Members Fitness dataset. Dropping individual parts and modifying them results in decreased performance. Dropping semi-supervision had the biggest impact, and the uncertainty weighting and Student-t part play a crucial role. The ablation study indicates that each component of RBDC plays an important role in the success of our proposed methods. Statistical significance was tested using the Wilcoxon (1945) Signed-rank test to compare across multiple sets, with the Holm correction applied for multiple testing. Our method, RBDC, attained statistically significantly higher scores on all of ARI, NMI, Accuracy, and Silhouette Score compared to baseline (*p* < 0.05), demonstrating its statistically significantly better multi-class clustering performance across the various metrics.

**Table 12:** Ablation results on RBDC components—Gym Members Fitness.

Variant	ARI	Macro-AUC	Comment
Full RBDC	0.428 ± 0.012	0.862 ± 0.010	Baseline configuration
Without Student-t (Gaussian decoder)	0.394 ± 0.014	0.838 ± 0.012	Gaussian decoder replaces Student-t
Without uncertainty weighting	0.371 ± 0.015	0.824 ± 0.013	Reduced robustness to noise
Without semi-supervision	0.349 ± 0.017	0.801 ± 0.015	Weaker alignment with structure
Without EM-step (SGD only)	0.378 ± 0.016	0.829 ± 0.014	Cluster params updated via SGD alone

Figure 6 shows the 2D latent space recovered by the proposed RBDC model. The horizontal axis is the Z_1_ dimension, and the vertical axis is the Z_2_ dimension. There are 4 visually discernible groups of points, suggesting that the model could learn 4 separate, well-defined clusters of latent variables. The points have been colored by the probability that they belong to their cluster label, and the color bar shows the corresponding probability scale. Light yellow regions represent “high-certainty” data—that is, samples for which the model predicted the labeled cluster with a high probability; and dark blues/purples represent “low-certainty.” The color bar spans approximately 0.25 to 0.82, consistent with the theoretical minimum membership probability of 1/K = 0.25 for K = 4 clusters, supporting the validity of the probability estimates. The majority of data points are tightly clustered around their centroid with high probability, and, in most cases, low-probability data appear at the fringes between clusters.

**Figure 6:**
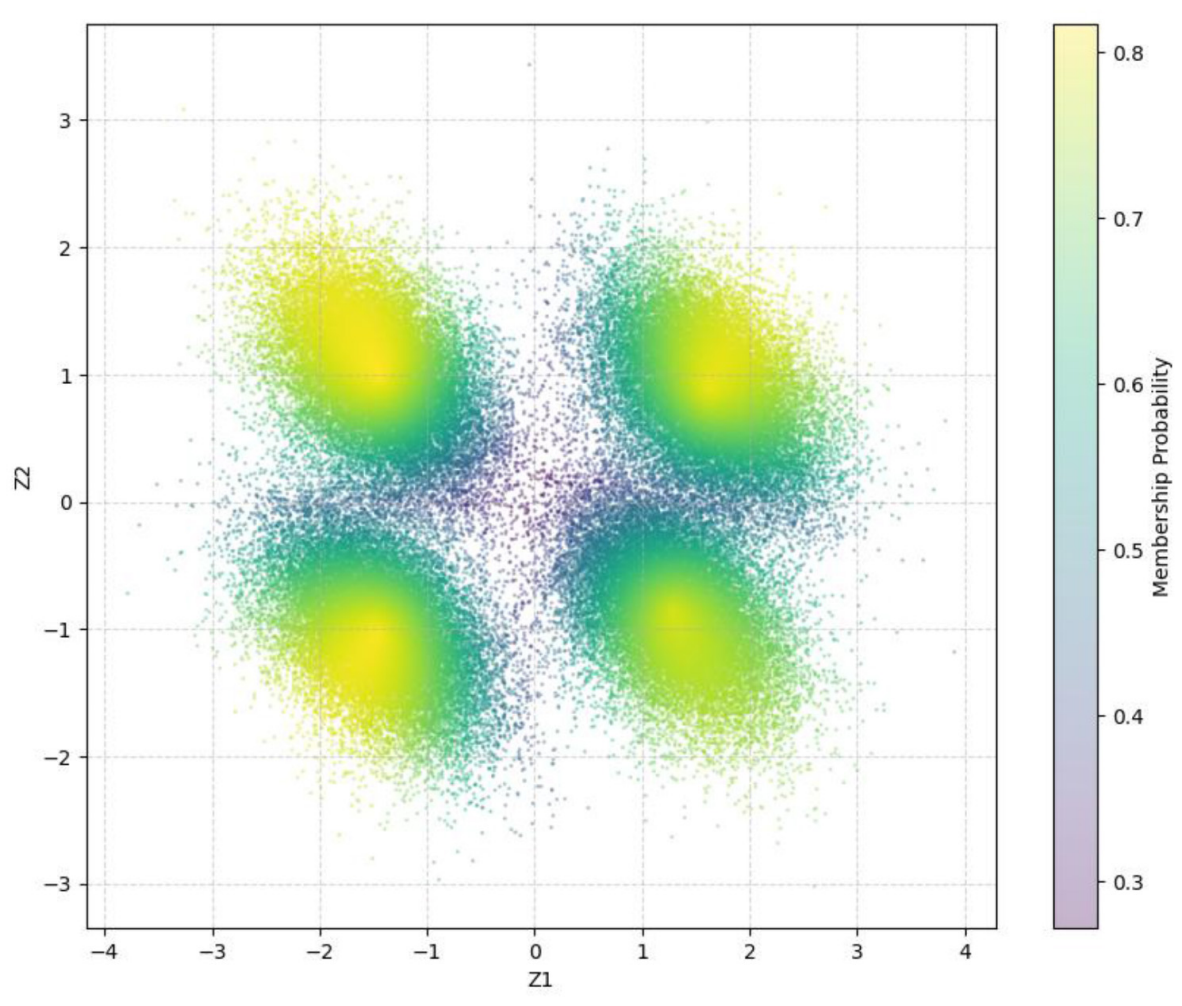
Latent space visualization colored.

### 4.3 Computational efficiency

To investigate scalability, training time, memory usage, and convergence behavior of RBDC, as well as the best baseline (VaDE), were reported for both datasets. Table 13 shows the time per epoch, wall-clock time, training epochs required to converge, training wall-clock time, and peak memory footprint for each setup.

**Table 13:** Training time and memory usage.

Dataset	Method	Time per epoch (s)	Convergence epoch	Total training time (min)	Peak memory (MB)
Adult	VaDE	8.7	142	20.6	1,284
Adult	RBDC	6.3	118	12.4	1,067
Fitness	VaDE	11.5	156	29.9	1,536
Fitness	RBDC	8.9	127	18.8	1,291

Table 13 compares the computational requirements of RBDC and VaDE in terms of training speed, convergence, and memory consumption. RBDC requires less time per epoch and reaches convergence in fewer epochs on both datasets. Consequently, its overall training time is lower than VaDE's while maintaining a smaller peak memory footprint. These results indicate that RBDC provides improved computational efficiency without substantially increasing resource requirements.

### 4.4 Baseline hyperparameter configurations

The hyperparameter search ranges and the selected values for each baseline method are summarized in Table 14. All hyperparameters were selected based on validation-set performance using the same experimental protocol as RBDC.

**Table 14:** Baseline hyperparameter configurations.

Method	Hyperparameter	Search range	Selected value
K-Means	Number of initializations	{10, 20, 50}	50
GMM	Number of initializations	{10, 20, 50}	50
GMM	Covariance type	{full, diag, tied}	full
DEC	Learning rate	{e^−4^, e^−3^, e^−2^}	e^−3^
DEC	Pretraining epochs	{50, 100, 200}	100
VaDE	Learning rate	{e^−4^, e^−3^, e^−2^}	e^−3^
VaDE	Pretraining epochs	{50, 100, 200}	100
HI-VAE	Latent dimension	{8, 16, 32}	16
M2	Learning rate	{e^−4^, e^−3^, e^−2^}	e^−3^
M2	Classification loss weight (α)	{0.1, 0.5, 1.0}	0.5
RBDC	Learning rate	{e^−4^, e^−3^, e^−2^}	e^−3^
RBDC	Latent dimension	{8, 16, 32}	16
RBDC	Batch size	{32, 64, 128}	64
RBDC	Number of training epochs	{100, 200, 300}	200
RBDC	Reconstruction loss weight	{0.1, 0.5, 1.0}	0.5

Table 14 shows the hyperparameter search ranges and the corresponding selected values for all baseline methods. For K-means and GMM, we searched over the number of initializations and for GMM, over the number of initializations as well as the covariance structures. For DEC and VaDE, we tuned learning rates and the number of pretraining epochs to find optimal optimization parameters, while for HI-VAE, we adjusted the latent space size and learning rates. Note that these values were chosen based on the performance on the validation set, using the exact same procedure and computational budget for each baseline method.

We then examine model calibration using AUC, Brier Score, and ECE values, as reported in Table 5. RBDC exhibits the best calibration result: AUC of 0.812 ± 0.007, Brier Score of 0.139 ± 0.006, and ECE of 0.058 ± 0.004, whereas the corresponding values of VaDE embeddings are 0.789 ± 0.009, 0.154 ± 0.007, and 0.074 ± 0.005, respectively. A lower Brier Score and ECE imply more reliable and better-calibrated probability outputs from RBDC, whilst a higher AUC indicates RBDC captures better discriminant properties of the data. These results provide further support for the merits of RBDC for reliable downstream predictions, owing to its uncertainty-aware optimization and probabilistic clustering mechanism.

## 5 Conclusion

To address noise, missing data, and limited labels in tabular datasets, this work introduced Robust Bayesian Deep Clustering (RBDC), a semi-supervised Bayesian framework. RBDC enhances clustering accuracy, resilience, and calibration by combining a robust Student-t decoder, label-informed Gaussian mixture priors, uncertainty-weighted learning, and hybrid EM–SGD optimization. Under low-label circumstances, experiments on the UCI Adult Income and Gym Members Fitness datasets showed steady improvements over current deep clustering techniques. Additionally, the system demonstrated adaptive cluster selection, automatically keeping only the number of clusters supported by the data. Semi-supervision is the main factor influencing clustering performance, while uncertainty weighting is the main factor influencing calibration improvements, according to ablation analysis. In summary, the results demonstrate that RBDC is a robust and effective framework for semi-supervised clustering of partially labeled, noisy, and incomplete tabular data.

For now, this evaluation is limited to tabular datasets; extension of this Student-t/GMM-prior formulation to data that is image or graph-structured—where the MLP encoder should be replaced by a convolutional or graph-based encoder, respectively—is a natural next research path.

## Data Availability

The original contributions presented in the study are included in the article/supplementary material, further inquiries can be directed to the corresponding author.
